# Are pediatricians familiar with hereditary angioedema?

**DOI:** 10.1016/j.waojou.2023.100783

**Published:** 2023-06-10

**Authors:** Herberto José Chong-Neto, Bárbara Padilha Aroni, Eli Mansour, Eliana Toledo, Faradiba Sarquis Serpa, Luisa Karla Arruda, Pedro Giavina-Bianchi, Solange Oliveira Rodrigues Valle, Caroline Guth de Freitas Batista de Moraes, Tatielly Kruk, Débora Carla Chong-Silva, Dirceu Solé, Luciana Rodrigues Silva, Anete S. Grumach, Nelson Augusto Rosário Filho, Régis de Albuquerque Campos

**Affiliations:** a Division of Allergy and Immunology, Complexo Hospital de Clínicas da Universidade Federal do Paraná, Curitiba, Brazil; b Clinical Immunology and Allergy, Department of Clinical Medicine, University of Campinas, Campinas, Brazil; c Serviço de Alergia e Imunologia Clínica do Departamento de Pediatria e Cirurgia Pediátrica da Faculdade de Medicina de São José do Rio Preto. São José do Rio Preto, Brazil; d Serviço de Asma, Alergia e Imunologia Clínica, Escola Superior de Ciências da Santa Casa de Misericórdia de Vitória, Vitória, Brazil; e Department of Medicine, Ribeirão Preto Medical School, University of São Paulo, Ribeirão Preto, Brazil; f Clinical Immunology and Allergy Division, Universidade de São Paulo, São Paulo, Brazil; g Departamento de Clínica Médica, Serviço de Imunologia, Hospital Universitário Clementino Fraga Filho, Universidade Federal do Rio de Janeiro, Rio de Janeiro, Brazil; h Division of Allergy, Clinical Immunology and Rheumathology, Department of Pediatrics, Federal University of São Paulo, São Paulo, Brazil; i Universidade Federal da Bahia, Divisão de Gastroenterologia Pediátrica, Salvador, Brazil; j Clinical Immunology, Centro Universitario FMABC, Santo André, Brazil; k Faculdade de Medicina da Universidade Federal da Bahia, Departamento de Medicina Interna e Apoio Diagnóstico, Programa de Pós-Graduação em Ciências da Saúde, Salvador, Brazil

**Keywords:** Angioedema, Hereditary, Pediatrics, Knowledge, Surveys Questionnaires

## Abstract

**Background:**

Hereditary angioedema (HAE) is an autosomal dominant disease characterized by recurrent episodes of subcutaneous or mucosal edema caused by excess bradykinin. The aim of the present study was to assess the knowledge of pediatricians about hereditary angioedema.

**Methods:**

An online survey with 12 HAE-related and 14 demographics-related questions was e-mailed to all pediatricians who were members of the Brazilian Society of Pediatrics (n = 17 145) once a week during the months of June and July 2021. The electronic questionnaire assessed clinical manifestations, diagnosis, and treatment of hereditary angioedema in children and adolescents.

**Results:**

Four hundred and fifty-five pediatricians responded to the questionnaire (2.6%), of whom 55 (12.1%) were board certified in Allergy and Immunology (A/I), while 400 (87.9%) were not (N-A/I). Three hundred and sixty-eight (80.9%) were female, 289 (55.7%) were under 50 years of age, 286 (62.9%) graduated from Medical School more than 10 years previously, 83 (18.2%) held an MSc/PhD degree, and 253 (55.6%) were living in the Southeast Region of Brazil. The median number of correct answers to the questions related to HAE among A/I was 7 out of 12 (58.3%), with median ranging from 4.5 to 8 correct answers, while for N-A/I it was 3 (25%), with median ranging from 2.5 to 4 correct answers (p < 0.001).

**Conclusion:**

Knowledge about HAE among Brazilian pediatricians, whether board certified in Allergy and Immunology or not, was unsatisfactory. HAE is a rare disease, largely unknown among physicians; therefore, increasing awareness may lead to improvement in diagnosis and treatment.

## Introduction

Hereditary angioedema (HAE) is an autosomal dominant disease characterized by recurrent episodes of localized, non-inflammatory, not pruritic, and asymmetric edema in the deep dermis, subcutaneous, or submucosa, affecting multiple organs as a result of overproduction of bradykinin.^1^^,^^2^ The estimated prevalence of HAE is 1:50.000 persons. No epidemiological studies estimating the prevalence of HAE in Brazil have been available, neither in adults nor the pediatric population. As a rare condition, HAE is unknown to many healthcare professionals and often underdiagnosed.^1^, ^2^, ^3^ Lack of appropriate treatment may lead to increased morbidity and mortality of the disease.^1^, ^2^, ^3^

Comparing 34 children with C1-INH-HAE with 64 healthy controls, children with symptomatic C1-INH-HAE demonstrated impaired HRQoL compared with healthy controls. HRQoL was affected by the frequency and site of C1-INH-HAE attacks and mostly in the school and physical domains.^4^ Brazilian patients with HAE demonstrated an impaired quality of life, as measured by the SF-36. The most affected domains were those related to vitality and social characteristics.^5^

The estimated mortality rate for untreated patients is 25–40%, with deaths mainly caused by laryngeal angioedema and subsequent asphyxiation.^1^ HAE patients had a mean of 4.4 medical evaluations before being correctly diagnosed, and 65% of them had at least 1 misdiagnosis.^3^

In an Iranian study, the median delay of diagnosis was 3 (range: 1.75–7.25) years, independent of family history.^6^ Results of a study involving 95 Brazilian children with HAE from 17 reference centers, revealed that the mean age at onset of symptoms was 3.3 years of age, whereas medical diagnosis was established only at 7.2 years of age, with 10% of these children undergoing abdominal surgical procedures, mainly appendicectomy, prior to diagnosis.^7^ In 32 patients from 23 families in India, the median age at onset of symptoms was 6.25 years (range 1–25 years), and the median age at diagnosis was 12 years (range 2–43 years). Life-threatening episodes of laryngeal edema were recorded and 1 disease-related mortality occurred in this cohort.^8^

A cohort of 433 patients from 46 families was evaluated in a prospective and retrospective study. Families were organized in clusters and were given a verbal autopsy report to arrange data collection for the deaths and analyze symptoms during life. Causes of death were classified as deaths from laryngeal edema (LE) or other causes. A total of 75 fatal events were evaluated. Only 10 of 75 patients were given the diagnosis of HAE before death, and the HAE diagnosis was made after death in 65 of 75 patients with a verbal autopsy report. The patients who did not receive an HAE diagnosis had more severe attacks that were responsible for the majority of the fatalities.^9^

Data on knowledge of physicians about HAE in children and adolescents are scanty. The aim of the present study was to evaluate the level of knowledge about HAE among pediatricians, specialists or not in Allergy and Immunology.

## Methods

We carried out an observational transversal three-part questionnaire study with 12 questions on clinical manifestations, diagnosis, and treatment of HAE in children and adolescents. An electronic questionnaire produced on Google Forms® platform (Google Inc., San Francisco, USA), not previously applied in a pilot study, was sent by e-mail to 17 145 pediatricians, members of the Brazilian Society of Pediatrics (BSP) during the months of June and July 2021.

The questionnaire was developed by members of the Brazilian Group for the Study of Hereditary Angioedema (GEBRAEH) with great experience in the field. The instrument has questions about demographic data, previous experience with HAE patients’ assistance and specific knowledge in HAE. All questions about HAE need to be agreed on by 90% of the 7 members who participated in the elaboration of the questionnaire.

All the questionnaires filled out by the pediatricians, who signed informed consent prior to responding, were included in the study (Appendix 1). For questions with more than one correct alternative, we considered as a correct answer only if all correct alternatives were marked.

The number of correct answers was compared in the groups of specialists in Allergy and Immunology (A/I) and non-specialists in Allergy and Immunology (N-A/I), and among those with less than 10 years and with 10 years or more since graduation from Medical School.

All data were transformed into an electronic Microsoft Excel® spreadsheet. Categorical and ordinal variables were expressed in frequency distribution, and χ^2^ test was used for comparison of proportions. Quantitative variables were expressed as median, minimum and maximum, and compared using Mann-Whitney test. For level of significance, an α < 0.05 was considered as significant. Statistical analyses were performed on StatPlus:mac® 5.9.5.0 (AnalystSoft Inc.2015 Walnut, CA, USA).

The present study was approved by the Complexo Hospital de Clínicas da Universidade Federal do Paraná Ethics Committee for Research in Humans (protocol number 45467921.1.0000.0096). All participants were not identified and signed informed consent.

## Results

From a total of 17 154 members of the Brazilian Society of Pediatrics, 455 (2.6%) responded to the questionnaire, including 400 (87.9%) pediatricians N-A/I and 55 (12.1%) pediatricians A/I. No significant differences were found regarding age, gender, geographical region, time since graduation from Medical School, type of practice, education level, and information received about HAE during Medical School or later, in the N-A/I and A/I groups. Self-assessment of knowledge about HAE proved to be higher in the A/I group, as compared to the N-A/I group (Table 1).

**Table 1:** Demographic characteristics of Brazilian pediatricians who responded the questionnaire

Demographic characteristics	A/I	N-A/I	p^a^
	n = 55 (12.1%)	n = 400 (87.9%)	
Age			
<30 years	3 (5.5)	52 (13)	0.2
30–39 years	22 (40)	120 (30)	
40–49 years	10 (18.2)	92 (23)	
50–59 years	12 (21.8)	62 (15.5)	
60 years or more	8 (14.5)	74 (18.5)	
Gender			
Female	42 (76.4)	326 (81.5)	0.36
Male	13 (23.6)	74 (18.5)	
Region			
Midwest	4 (7.3)	21 (5.3)	0.69
Northeast	5 (9.1)	58 (14.5)	
North	3 (5.5)	20 (5)	
Southeast	34 (61.8)	219 (54.8)	
South	9 (16.4)	82 (20.5)	
Time since graduation from Medical School			
Less than 10 years	19 (34.5)	150 (37.5)	0.67
10 years or more	36 (65.5)	250 (62.5)	
Type of practice			
Healthcare	46 (83.6)	363 (90.8)	0.1
Academic	9 (16.4)	37 (9.3)	
Education level			
A/I Fellowship/Board certified	43 (78.2)	329 (82.2)	0.46
MSc/PhD	12 (21.8)	71 (17.8)	
Knowledge about HAE during Medical School			
No	41 (74.5)	301 (75.3)	0.33
Knowledge about HAE during A/I Fellowship or Pediatric Residency			
No	38 (69.1)	230 (57.5)	
Knowledge about HAE during after Fellowship or Pediatric Residency			
No	3 (5.5)	42 (10.5)	
Self-assessment of knowledge			
Regular/insufficient	17 (30.9)	340 (85)	0.001
Great/good	38 (69.1)	22 (5.5)	
Do not know	0	1 (0.3)	
Care of someone with HAE			
No	11 (20)	210 (52.5)	0.01
Yes	42 (76.3)	123 (30.7)	
Do not remember	2 (3.7)	67 (16.8)	

a χ^2^ test.

The median number of correct answers among A/I, independent of time since graduation from Medical School, was 7/12 correct answers (58.3%), whereas for N-A/I the median number of correct answers was 3/12 (25%) (p < 0.001). However, analyzing the median of the number of correct answers, no significant difference was observed among A/I with <10 years [7 (range 2–10)] versus ≥10 years [7 (range 3–10)] of graduation, p = 0.34, and among N-A/I with <10 years [3 (range 0–9)] versus ≥10 years [3 (range 0–10)] of graduation, p = 0.1. There was a significant difference in median number of correct answers among A/I versus N-A/I pediatricians graduated from Medical School <10 years living in the North, Northeast, and Southeast regions, with higher median number of correct answers among A/I. The study also showed a significant difference in median number of correct answers among A/I versus N-A/I pediatricians graduated from Medical School ≥10 years living in the Southeast and South regions, with higher median number of correct answers among A/I (Table 2).

**Table 2:** Median number of correct answers among pediatricians with <10 years and ≥10 years of graduation from Medical School

Regions	A/I (<10 years)	N-A/I (<10 years)		A/I (≥10 years)	N-A/I (≥10 years)	
	n (range)	n (range)	p	n (range)	n (range)	p
Midwest	–	3 (1–7)	∗	4.5 (3–10)	3.5 (1–8)	0.18
Northeast	6 (5–9)	4 (2–8)	0.01	5 (4–6)	3 (1–8)	0.11
North	7 (6–8)	2.5 (1–5)	0.03	7^(7)^	3 (2–7)	#
Southeast	7 (2–10)	3 (0–10)	0.001	6 (3–9)	3 (1–9)	<0.001
South	7 (7–7)	3 (0–10)	0.05	8 (7–9)	3 (1–7)	<0.001

∗ It was not possible to calculate p-value due lack of A/I among responders from Midwest region.

# Not possible to calculate (only 1 specialist from the North region).

Questions were subdivided into 3 areas: clinical manifestations, diagnosis, and treatment of HAE. Pediatricians A/I correctly answered questions on clinical manifestations and diagnosis, but not on treatment (Table 3).

**Table 3:** Median number of correct answers within the subareas of the questionnaire of clinical manifestations, diagnosis and treatment of HAE

	A/I n (%)	N-A/I n (%)	p
Clinical manifestations	39 (70.9)	97 (24.2)	0.008
Diagnosis	52 (94.5)	287 (71.7)	0.005
Treatment	14.5 (26.3)	12.5 (3.1)	0.33

## Discussion

HAE is a rare disabling disease, and early diagnosis and proper treatment are vital. In this study, we assessed, through an electronic questionnaire, the level of knowledge about HAE among pediatricians N-A/I and A/I, fulfilling a gap in information regarding pediatricians’ understanding of HAE diagnosis and treatment in Brazil.

The delay in diagnosis of HAE has been reported as 11–20 years. The onset of symptoms of HAE with C1–INH deficiency (HAE-C1-INH) usually occurs in childhood or adolescence; however, most patients are not diagnosed until they reach adulthood. The disease is frequently misdiagnosed as an allergic condition, appendicitis, or biliary disorders .^10^^,^^11^

In the Icatibant Outcome Survey (IOS), patients recorded any misdiagnosis received prior to the diagnosis of HAE-C1-INH. From a total of 418 participants with HAE-C1-INH type I or II who provided information on this matter, 44.3% had 1 or more previous misdiagnosis.^11^ As a result, the delay in diagnosis was 13.3 years on average. The low level of knowledge contributes to this delay, not only in diagnosis but also in proper treatment as well, leading to impairment in quality of life and increase in mortality.^10^^,^^11^

The rate of participation in this study was low as observed in another study on the management of HAE among US doctors, where 172 voluntarily completed an online survey developed by physician-investigators. A wide variability was reported in the treatment of patients with HAE in United States, because the survey was completed shortly after approval of additional HAE therapies by the US Food and Drug Administration (FDA).^12^ There was a significant contribution by female pediatricians and residents in the Southeast region, which is in line with the demographic characteristics of physicians with training in pediatrics in Brazil.^13^ Nonetheless, no difference in the demographic distribution was observed among pediatricians who were board-certified and not board-certified in Allergy and Immunology, and regarding age, gender, geographical region, time since graduation from Medical School, type of practice, education level, or information about HAE received during medical training or medical practice.

N-A/I self-classified their knowledge about HAE as regular or insufficient, while A/I presented a better self-assessment, probably because they have treated more HAE patients during and/or after A/I Fellowship, compared to N-A/I. Nevertheless, some of N-A/I acknowledged they have treated patients with HAE; however, we cannot rule out that some of these patients may have other types of angioedema, misdiagnosed as HAE.

In the present study, no significant difference was detected for knowledge about the disease when considering time since graduation from Medical School. However, a study carried out in Turkey in 2015, by which 155 internists answered a 20-item questionnaire on HAE during the National Congress of Internal Medicine, showed that awareness about the disease was higher among younger doctors (35.9 years-old versus 45.7 years-old).^14^ In that study, the average time from HAE onset of symptoms to diagnosis was 26 years.^14^

Our understanding of hereditary angioedema has grown exponentially over the past 2 decades due to better comprehension in pathophysiology and the discovery of new genetic variants as cause of the disease. It was expected that physicians with longer time since graduation from Medical School could have more limited knowledge and could perform less than younger doctors. This situation was not confirmed in the present study, when we compared performance on the questionnaire of pediatricians with less than 10 years versus those with 10 or more years since graduation from Medical School. However, we have not analyzed their training courses and the number of treated HAE patients by each physician.

The highest median number of correct answers among A/I pediatricians with 10 or more years since graduation from Medical School living in the South and Southeast regions of Brazil may be explained by greater concentration of reference centers and pioneering in the care of children with HAE, allied to academic excellence. However, more recently an increase in the presence of specialist and reference centers in the North and Northeast regions, in addition to the South and Southeast, has been observed.

Although the number of correct answers by responders from the Southeast, which was the region with the highest number of participants, was higher, the results were still far from being satisfactory, with a total of correct answers close to 50% even among A/I pediatricians, indicating limited knowledge about the disease.

Questions with more than 1 correct alternative were used to evaluate the complete knowledge about HAE. For questions on clinical manifestations and diagnosis, the overall performance was superior among A/I as expected; however, questions on treatment showed worse results among A/I and N-A/I, possibly due to low familiarity with newer therapeutical options, that have recently been approved by the Brazilian Health Agency, or misdiagnosis with conditions such as mast cell-induced angioedema, resulting in ineffective treatment.

Studies evaluating medical knowledge about rare diseases, or even more common diseases, are scarce. A multicentric study conducted in Russia and Ukraine tested doctors’ knowledge about community acquired pneumonia, a non-rare disease, and reported a mean of 49.6% correct answers by 255 participants.^15^ Similarly, in an evaluation on anaphylaxis in emergency rooms, the level of medical knowledge was 44.3%.^16^ These levels of medical knowledge are far below expected, which also occurs in HAE. In the study from Turkey with internists, 93.5% were aware of HAE and 41.9% had treated at least one patient with hereditary angioedema^14^.

Limitations of our study include the fact that we have not used a standardized and validated questionnaire, because none were available at the time and some questions were subjective (question 13). However, the instrument was developed by a well-established and experienced study group in HAE. Our instrument included objective questions which enabled us to assess the low level of knowledge about HAE among A/I and N-A/I pediatricians. The questionnaire conducted virtually and with a voluntary, not motivated, participation, may have induced a bias toward higher engagement of pediatricians with interest in this specific disease.

Brazilian pediatricians presented, in general, a low level of knowledge about HAE, even when A/I board-certification were considered. Based on the present study, actions are needed to raise awareness about HAE in order to reduce time for diagnosis and initiation of a proper treatment, improving patients’ quality of life and reducing disease mortality.

## Abbreviations

A/I group, pediatrics with Allergy and Immunology certification; BSP, Brazilian Society of Pediatrics; C1–INH-HAE, Hereditary angioedema with C1 inhibitor deficiency; GEBRAEH, Brazilian Group for the Study of Hereditary Angioedema; HAE, Hereditary angioedema; HRQoL, Health related quality of life; N-A/I group, pediatrics with no Allergy and Immunology certification; SF-36, Short form 36.

## Acknowledgments

The authors would like to thank Brazilian Society of Pediatrics for agree to participate and send all e-mail to the pediatricians during the study period.

## Financial support

There are no financial support.

## Availability of data and materials

Not applicable, review paper only.

## Author's contribution

**Régis de Albuquerque Campos**: Conceptualization; methodology; validation; visualization; writing –review and editing. **Eli Mansour**: Conceptualization; writing –original draft; methodology; validation; visualization; writing –review and editing. **Eliana Toledo**: Conceptualization; methodology; validation; visualization; writing –review and editing. **Faradiba Sarquis Serpa**: Conceptualization; methodology; validation; visualization; writing –review and editing. **Luisa Karla Arruda**: Conceptualization; methodology; validation; visualization; writing –review and editing. **Pedro Giavina-Bianchi**: Conceptualization; methodology; validation; visualization; writing –review and editing. **Solange Oliveira Rodrigues Valle**: Conceptualization; methodology; validation; visualization; writing –review and editing. **Bárbara Padilha Aroni:** Investigation; writing –original draft; visualization; formal analysis; data curation; supervision. **Caroline Guth de Freitas Batista de Moraes**: Investigation; writing –original draft; formal analysis. **Tatielly Kruk:** Investigation; writing –original draft; formal analysis. **Débora Carla Chong-Silva:** Investigation; writing –original draft; writing –review and editing. **Dirceu Solé:** Investigation; writing –original draft; writing –review and editing. **Luciana Rodrigues Silva**: Investigation; writing –original draft; writing –review and editing. **Anete S. Grumach**: Conceptualization; investigation; writing –original draft; methodology; validation; visualization; writing –review and editing. **Nelson Augusto Rosário Filho**: Conceptualization; investigation; writing –original draft; methodology; validation; visualization; writing –review and editing; formal analysis; data curation; supervision. **Herberto José Chong-Neto:** Conceptualization; investigation; writing –original draft; methodology; validation; visualization; writing –review and editing; formal analysis; data curation; supervision.

## Ethics approval and consent to participate

The present study was approved by the Complexo Hospital de Clínicas Ethics Committee for Research in Humans (protocol number 45467921.1.0000.0096).

## Consent for publication

All authors agreed for this work to be published in the World Allergy Organization Journal.

## Declaration of competing interest

Nothing to declare.

## Appendix 1

Assessment of knowledge on Hereditary Angioedema among Brazilian pediatricians.

You are being invited to take part in the study “Assessment of knowledge on Hereditary Angioedema among Brazilian pediatricians” for being a pediatrician, through a questionnaire with an approximate duration of 5 min. This is an initiative of the Scientific Department of Allergy of the Brazilian Society of Pediatrics in conjunction of the Federal University of Paraná. The objective of this study is to assess the knowledge about Hereditary Angioedema (HAE) types I and II among pediatricians. The information collected here will be of special importance to create actions to expand and disseminate knowledge on Hereditary Angioedema among Brazilian pediatricians. Your participation in this study will consist in answering an online questionnaire on medical knowledge about HAE types I and II.

**1. Are you board-certified in Allergy and Immunology?**
No
Yes
**2. How old are you?**
Less than 30 years old
Between 30 and 39 years old
Between 40 and 49 years old
Between 50 and 59 years old
More than 60 years old
**3. Gender:**
Female
Male
**4. In which Brazilian state do you practice pediatrics?**
**5. For how many years have you been practicing pediatrics?**
Less than 5 years
Between 5 and 9 years
Between 10 and 19 years
Between 20 and 29 years
30 years or more
**6. Where does the doctor work most of the time? (select one or more alternatives)**
Private office
Private hospital
Private university
Basic health unit
Public hospital
Public university
**7. How do you best define your main activity in your workplace?**
Assistance
Graduation teacher
Postgraduation *latu sensu* teacher
Master/doctorate mentor
Health management
**8. What is your education level?**
Medical residency/Board certified
Master (Msc.)
Doctorate (Ph.D.)
**9. Did you learn anything about HAE during your medical graduation?**
Yes
No
**10. Did you learn anything about HAE during your residency/specialization in pediatrics?**
Yes
No
**11. After you completed residency have you heard or learned anything about HAE?**
Yes
No
**12. When was the last time you took part in a short-term updating course that addressed HAE in its program?**
During the pandemic
Up to two years
More than two years
I don't remember
I never took part
**13. How do you evaluate your knowledge on Hereditary Angioedema (HAE)?**
Excellent
Good
Regular
Insufficient
I don't know how to evaluate
**14. Have you ever took care of any patient or Family member of a patient with a diagnosis of HAE?**
No
Yes, I took care of a patient
Yes, I took care of a Family member of a patient
Yes, I took care of a patient and a family member of a patient
I don't remember if I took care of a patient and a family member of a patient

**MEDICAL KNOWLEDGE ON HEREDITARY ANGIOEDEMA**

**15. Which of the following signs and symptoms are suggestive of HAE? (you can mark as many options as necessary)**
Recurrent abdominal pain
Recurrent angioedema
Responsive to antihistamines
Recurrent urticaria and angioedema
Responsive to epinephrine
**16. What type of genetic inheritance is linked to HAE?**
Autosomal dominant
Autosomal recessive
X-linked
Type of inheritance not yet known
**17. Symptom onset of HAE usually occur:**
In the first year of life
Between 1 and 10 years of age
Between 11 and 20 years of age
Over 20 years of age
There no predilection for age group
**18. In children and adolescents, which is/are the site(s) of HAE attacks: (you can mark as many options as necessary)**
Airways
Face
Chest
Abdomen
Joints
**19. Which are the triggers for HAE attacks in childhood and adolescence? (you can mark as many options as necessary)**
Insect bites
Foods
Viral infections
Surgeries
Alcohol consumption
**20. Which hormone is linked to triggering HAE symptoms?**
Androgen
Estrogen
Progesterone
FSH
**21. Is considered as a screening test for the diagnosis of HAE:**
CH50
C3
C4
C1-inhibitor (C1–INH)
**22. The absence of a family history of HAE rues out the diagnosis?**
Yes
No
**23. Which laboratory test confirms the diagnosis of HAE types 1 and 2?**
Complement component 3 measurement (C3)
Complement component 4 measurement (C4)
CH50 measurement
Quantitative and qualitative measurements of C1-inhibitor.
**24. What is the main mediator related to HAE?**
Acetylcholine
Bradykinin
Prostaglandin
Histamine
**25. Which of the following options are treatment for HAE attack?**
Icatibant
Fresh frozen plasma
Epinephrine
C1–INH concentrate
Tranexamic acid
**26. Which of the following options are treatment for HAE prophylaxis?**
Androgens
C1–INH concentrate
Tranexamic acid
Lanadelumab
